# Feasibility of adenosine stress cardiovascular magnetic resonance perfusion imaging in patients with MR-conditional transvenous permanent pacemakers and defibrillators

**DOI:** 10.1186/s12968-021-00842-0

**Published:** 2022-01-13

**Authors:** Anna Giulia Pavon, Alessandra Pia Porretta, Dimitri Arangalage, Giulia Domenichini, Tobias Rutz, Sarah Hugelshofer, Etienne Pruvot, Pierre Monney, Patrizio Pascale, Juerg Schwitter

**Affiliations:** 1 Division of Cardiology, Cardiocentro Ticino Institute, Ente Ospedaliero Cantonale, Via Tesserete, 48, 6900 Lugano, Switzerland; 2 Cardiovascular Department, Division of Cardiology, Lausanne University Hospital (CHUV), Lausanne, Switzerland; 3 Cardiac Magnetic Resonance Center of the CHUV (CRMC), Lausanne University Hospital, Lausanne, Switzerland; 4 Faculty of Biology and Medicine, University of Lausanne (UniL), Lausanne, Switzerland; 5 Cardiology Department, AP-HP, Bichat Hospital and Université de Paris, Paris, France

**Keywords:** Cardiovascular magnetic resonance, Stress test, Adenosine, Implantable device, Pacemaker, Implantable cardioverter defibrillator, MRI conditional, Safety

## Abstract

**Background:**

The use of stress perfusion-cardiovascular magnetic resonance (CMR) imaging remains limited in patients with implantable devices. The primary goal of the study was to assess the safety, image quality, and the diagnostic value of stress perfusion-CMR in patients with MR-conditional transvenous permanent pacemakers (PPM) or implantable cardioverter-defibrillators (ICD).

**Methods:**

Consecutive patients with a transvenous PPM or ICD referred for adenosine stress-CMR were enrolled in this single-center longitudinal study. The CMR protocol was performed using a 1.5 T system according to current guidelines while all devices were put in MR-mode. Quality of cine, late-gadolinium-enhancement (LGE), and stress perfusion sequences were assessed. An ischemia burden of ≥ 1.5 segments was considered significant. We assessed the safety, image quality and the occurrence of interference of the magnetic field with the implantable device. In case of ischemia, we also assessed the correlation with the presence of significant coronary lesions on coronary angiography.

**Results:**

Among 3743 perfusion-CMR examinations, 66 patients had implantable devices (1.7%). Image quality proved diagnostic in 98% of cases. No device damage or malfunction was reported immediately and at 1 year. Fifty patients were continuously paced during CMR. Heart rate and systolic blood pressure remained unchanged during adenosine stress, while diastolic blood pressure decreased (p = 0.007). Six patients (9%) had an ischemia-positive stress CMR and significant coronary stenoses were confirmed by coronary angiography in all cases.

**Conclusion:**

Stress perfusion-CMR is safe, allows reliable ischemia detection, and provides good diagnostic value.

**Supplementary Information:**

The online version contains supplementary material available at 10.1186/s12968-021-00842-0.

## Introduction

Cardiovascular magnetic resonance (CMR) plays an increasingly important role in ischemia detection with class I/level A, and class IIa recommendations in the current European and North American guidelines [1, 2]. For many years, the presence of an implantable electronic device was considered an absolute contraindication for magnetic resonance (MR) scanning [3, 4] until the advent of MR-conditional devices and the documentation of their safety [5–9]. Thus, MR-conditional permanent pacemakers (PPM) and implantable cardioverter-defibrillators (ICD) are now compatible with routine CMR examinations [10]. However, the application of adenosine stress-CMR in patients implanted with MR-conditional devices has only been investigated in a limited number of studies [11, 12]. Pezel et al. showed that patients with PPMs without inducible ischemia or late gadolinium enhancement (LGE) had a very low cumulative rate (0.9%) of major adverse cardiovascular events (MACE) over a 7-year follow-up [13] demonstrating the good prognostic value of stress perfusion CMR. Despite these results, the use of stress perfusion CMR in this population remains limited, mainly due to the possible occurrence of artefacts related to the metallic components of the devices and to the risk of adenosine interaction with cardiac pacing. It is worth mentioning that asynchronous pacing above resting heart rate (HR) during adenosine stress CMR as suggested by Klein-Wiele et al. was not found to interfere with the intrinsic cardiac rhythm [12, 13]. Although patients with cardiac conduction disorders may frequently suffer from underlying or concomitant coronary artery disease (CAD) [14], only a limited number of patients with atrioventricular (AV)-block requiring continuous pacing during adenosine stress-CMR have been reported (< 8 patients) [11–13].

In the present study, we sought to assess the safety of stress perfusion CMR in patients requiring pacing during adenosine stress, to evaluate image quality and to study the potential interferences of the magnetic field with PPM and ICD devices immediately after the CMR scan and at 1-year of follow-up. As a secondary objective, we evaluated the diagnostic performance of stress perfusion CMR during follow-up in patients with these devices.

## Methods

### Study population

We conducted a retrospective single-center analysis between August 2013 and March 2021 on a cohort of consecutive patients with implantable cardiac devices who were entered into the prospective CMR registry of our institution (CMR Center of Lausanne University Hospital, Lausanne, Switzerland). All patients were referred for adenosine stress perfusion CMR because of suspected or known CAD. Detailed medical history and the symptomatic status were recorded at the time of the CMR examination. Exclusion criteria were: (1) age < 18 years; (2) contraindication to CMR; (3) contraindication to adenosine (severe asthma or chronic obstructive pulmonary disease); (4) known allergy to gadolinium-based contrast medium; and (5) renal insufficiency defined by an estimated glomerular filtration rate < 30 ml/min/1.73 m^2^. All patients provided written informed consent and the study was approved by the local ethics committee.

### Patient follow-up and clinical outcome

Patients’ follow-up and clinical outcome were collected from medical records and/or from direct contact with the referring cardiologists.

The primary endpoint was (1) the safety of CMR in patients PPM dependent during vasodilator stress, (2) the evaluation of image quality of cine, LGE, and perfusion acquisitions based on modified criteria as published by Klinke et al. [15] in the presence of an electronic cardiac devices (PPMs or ICDs) Secondary aims were: (1) the presence of significant coronary artery stenosis on coronary angiography and its concordance with the detected stress perfusion defects on CMR, and the occurrence of MACE during a follow-up of at least 6 months and (2) the possible immediate and long-term interference of the magnetic field with the implantable device by measuring battery status, lead impedance, pacing capture thresholds, and sensing amplitudes. MACE was defined as cardiovascular (CV) death or non-fatal myocardial infarction (defined by detection of a rise and/or fall of cardiac troponin (cTn) values with at least one value above the 99th percentile of the upper limits of normal with at least one of the following between symptoms of acute myocardial ischaemia, new ischaemic electrocardiogram (ECG) changes, development of pathological Q waves or imaging evidence of new loss of viable myocardium or new regional wall motion abnormality in a pattern consistent with an ischaemic aetiology).

### PPM and ICD programming

CMR was performed more than 6 weeks after device implantation in all patients as recommended [4]. Participating patients arrived at the hospital 30 min before the CMR examination. Device status was documented, and the frequency of pacing episodes were recorded prior to the CMR. All devices were set to MR-safe mode according to manufacturers’ instructions. If an ICD was in place, pre-CMR programming was performed in the presence of the medical staff with continuous ECG monitoring. Patients with > 1% of atrial or ventricular pacing were set to continuous pacing in asynchronous mode (at 10 bpm above resting HR) irrespective of their actual rhythm in order to avoid possible asystole or bradycardia during the adenosine stress. VOO mode was selected in patients with continuous high grade AV block and DOO mode in patients with intermittent AV block or sick sinus syndrome. All devices were reprogrammed to previous device settings immediately after the CMR examination, and evaluated at 1 year.

### CMR protocol

ECG-gated stress CMR was performed using a 1.5 T CMR unit ( MAGNETOM Aera or Sola, Siemens Healthineers, Erlangen-Germany) with a 32-channel phased-array surface receiver coil. Two separate cubital venous cannulas were respectively used for adenosine and gadolinium-based contrast medium (GBCA) administration. Cine images were acquired using either a breath-hold balanced steady-state free precession (bSSFP) or a fast gradient echo (GRE) pulse sequence in long-axis (2-chamber, 3-chamber and 4-chamber) and short-axis views (8 mm slices, 2 mm gap, 10–15 slices). The decision to use GRE sequences was based on scout images in case of the presence of metallic artefacts and if the distance between the generator and the heart was < 7 cm. Stress perfusion imaging was performed using a standard GRE-based pulse sequence [16] which acquires 4 short-axis slices every 2 RR intervals to cover the left ventricle (LV). A preparatory acquisition of 30 frames (= 60 heart beats) was performed before gadolinium administration to detect potential artefacts and to subsequently adapt image orientation and/or field-of-view. Adenosine was administered as a 3-min infusion of 140 mg/kg/min. First-pass perfusion imaging was carried out with an IV bolus of a gadolinium (0.1 mmol/kg body weight gadobutrol, Gadovist®, Bayer Healthcare, Berlin, Germany) at an injection rate of 5 ml/sec. After injection of a second bolus of 0.1 mmol/kg of gadolinium and a 10–15 min waiting time, LGE images were acquired using a 2D breath-hold phase-sensitive segmented inversion-recovery fast gradient echo-based pulse sequence in the same orientations as the cine acquisitions. Inversion-time was individually optimized to null normal myocardium. Detailed table with CMR parameters is presented in Additional file 1: Table S1.

Patients were monitored during the entire CMR examination with continuous ECG surveillance and visual supervision by an experienced cardiologist with CMR level 3 certification provided by the European Society of Cardiology [17]. HR and blood pressure (BP) were recorded at baseline and every minute during adenosine infusion. The supervising cardiologist noted any arrhythmias, competitive pacing episodes, or bradycardias during the adenosine infusion.

### Image analysis

LV volumes and function were quantified on the short-axis cine stack applying Simpson’s rule (Argus VF or syngoVia software, Siemens Healthineers). Stress perfusion CMR images were evaluated according to the American Heart Association 17-segment model (excluding the apical segment 17) [18]. The perfusion images were analyzed visually by applying the previously described criteria [14, 16, 19]. A myocardium appearing dark for ≥ 3 frames (i.e. ≥ 6 heart beats) and a perfusion defect involving ≥ 1/3 of the LV wall thickness (i.e. > 1 pixel) and ≥ 50% of the circumferential extent of the segment defined ischemia. A segment was considered ischemic if more than a half of its extension was affected by the perfusion deficit. The presence of myocardial LGE was visually evaluated and its extent was reported as the number of LGE positive segments (according to the 17 segments model) [18]. The overall quality of the exams was graded taking into account 35 different parameters already validated by Klinke et al. [15]. The following items were assessed in the scoring system: LV coverage, the presence of artifacts such as wrap around, respiratory and/or cardiac ghost, image blurring or mis-triggering, metallic artifacts, shimming artifacts and signal loss. They were evaluated in cine images, as well as on LGE and first-pass perfusion sequences. In case of first-pass perfusion, in-plane spatial resolution, acquisition window and patient preparation were also considered. Considering a score from 0 to 64 points, quality was graded as non-diagnostic (grade 4, points from: 48–64), poor (grade 3, points from: 31–47), moderate (grade 2, points from: 16–30), or good (grade 1, points from: 0–15). A scoring of “Poor”, “Moderate” and “good” lead to an overall diagnostic CMR examination. The “splenic switch-off” was evaluated by using a visual comparison of the splenic tissue contrast enhancement and the myocardium on whichever of the 4 stress perfusion short-axis sections the spleen was seen best. Perfusion was graded as switched off when during stress perfusion the signal intensity was the same as the myocardium [20].

### Statistical analysis

Continuous variables were expressed as mean ± SD or median [interquartile range] where appropriate, and categorical variables as numbers and percentages (n, %). Continuous variable differences were analyzed with the independent-sample Student’s t test in case of a normal distribution or with the Mann–Whitney test in case of non-parametric distribution. Comparison of categorical variables was performed with the χ2 test or ANOVA. All statistical analyses were performed using SPSS (version 16.0, Statistical Package for the Social Sciences, International Business Machines, Inc., Armonk, New York, USA). A p < 0.05 was considered statistically significant.

## Results

### Baseline clinical characteristics

**Fig. 1:**
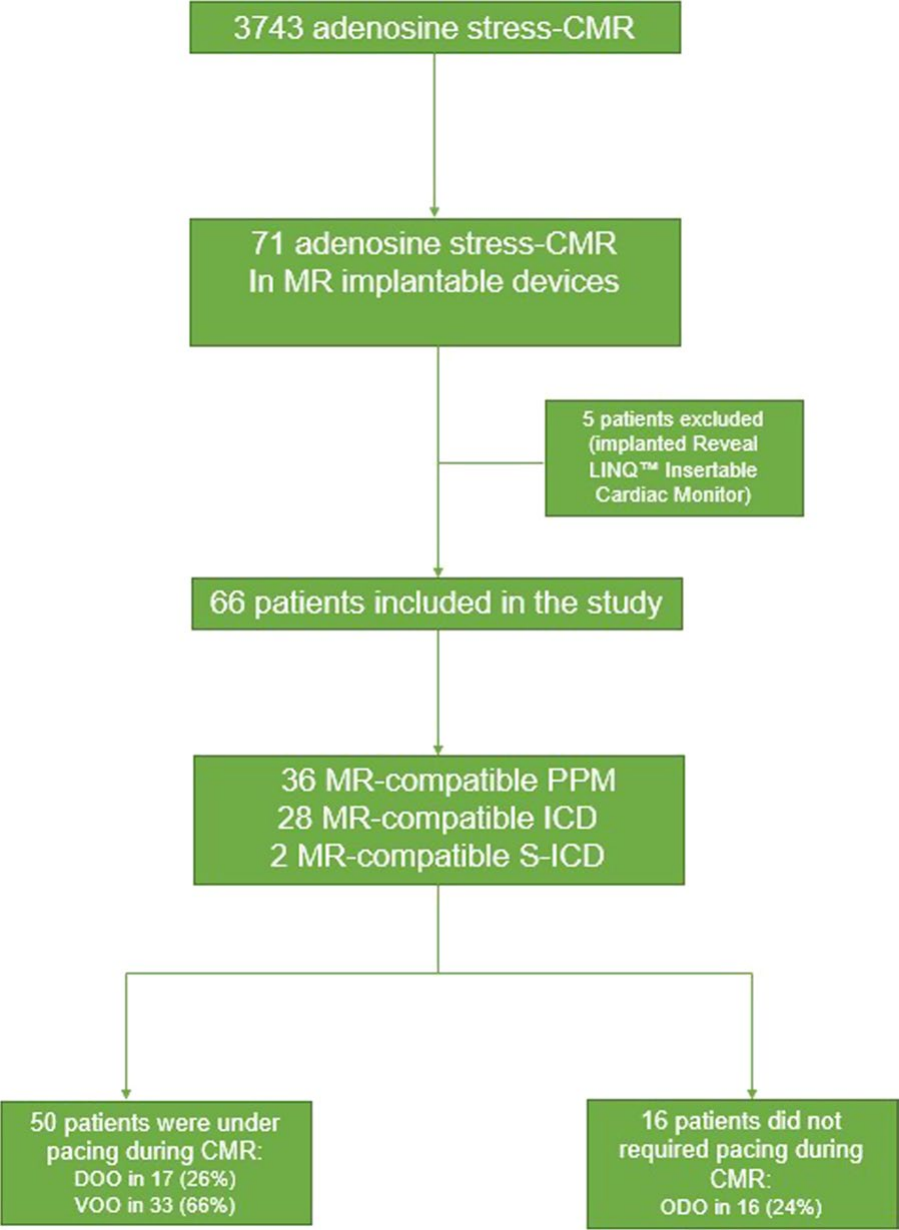
study flowchart

Between August 2013 and March 2021 a total of 3743 adenosine stress-CMR examinations were performed at our CMR centre. A total of 71 patients with implantable electronic cardiac devices underwent an adenosine stress-CMR examination. Five patients with an implanted Reveal LINQ™ Insertable Cardiac Monitor (Medtronic, Dublin, Ireland) were excluded from the analysis (Fig. 1). Among the remaining 66 patients, 36 were implanted with a transvenous PPM and 30 with an ICD (Fig. 1). Two patients underwent two stress perfusion CMR examinations yielding a total of 68 examinations. Among the 36 patients with a transvenous PPM, clinical indications were sinus node dysfunction in 11 patients (22%), second or third degree AV bloc in 20 patients (40%) and bradyarrhythmia with atrial fibrillation in 5 patients (10%). ICD indications were primary and secondary prevention in 28 (93%) and 2 patients (7%), respectively. Among patients with an ICD, 14 patients (50%) were paced by the device either because of a second or third degree atrio-ventricular block (10 patients, 36%) or sinus node dysfunction (4 patients 8%). Two patients (4%) were implanted with a subcutaneous ICD (S-ICD). Baseline characteristics are presented in Tables 1 and 2.

**Table 1:** Baseline clinical characteristics

	N	%
Total population	71	
Female	17	26
Hypertension	47	71
Diabetes	11	17
Hyperlipidemia	33	50
Smoking	14	21
Previous Ischemic Heart Disease	22	33
STEMI	6	27
NSTEMI	2	9
Elective PCI	9	41
CABG	5	23
Angina	23	35
CCS class I	17	74
CCS class II	6	26
CCS class III	0	0
CCS class IV	0	0
Dyspnea	17	26
NYHA class I	1	5
NYHA class II	11	65
NYHA class III	4	23
NYHA class IV	0	0
Ventricular Arrhythmia	26	39

STEMI, ST-elevation myocardial infarction; NSTEMI, Non-ST-elevation myocardial infarction, PCI, percutaneous coronary intervention; CABG, Coronary artery bypass grafting; NYHA, New York Heart Association; CCS, Canadian Cardiovascular Society

**Table 2:** MR-conditional devices characteristics

	N	%
Pacing Function Indication		
High degree AV block	30	60
Sinus node dysfunction	15	30
Bradyarrhythmia in AF	5	10
ICD Indication		
Primary prevention	28	93
Secondary prevention	2	7
Patients paced during CMR	50	76
Type of Pacing during CMR		
DOO	17	26
VOO	33	66
ODO	16	24

AV, atrioventricular; AF, atrial fibrillation; ICD, implantable cardio defibrillator; CMR, cardiovascular magnetic resonance

### Device setting and integrity

Among the 66 patients, 38 patients were PPM-dependent (defined as a HR < 30 bpm and > 95% of atrial or ventricular stimulations), while 12 patients (18%) presented a percentage of atrial or ventricular stimulation > 1%. Of the 50 patients set to pacing during the CMR examination, devices were set to DOO and VOO in 17 and 33 patients, respectively. In the remaining 16 patients (24%) who were not paced during the CMR examination (< 1% of atrial and ventricular stimulation), the device was an ICD and the PPM function was programmed to ODO. In all paced patients, the HR was programmed at least 10 bpm above resting HR yielding a range of 65 to 90 bpm. No competitive atrial or ventricular stimulation was observed during the CMR examinations.

Device integrity was not compromised by the CMR examination. Pacing capture thresholds, sensing amplitudes, lead impedance, and battery voltage remained unchanged pre- and post-CMR in both, atrial and ventricular leads. No patient had a pacing capture threshold increase > 0.5 V for the right atrial (RA) or right ventricular (RV) leads [9]. No changes were noted at 1-year, including no variation > 0.5 V in the RA or RV leads pacing capture thresholds. Table 3 summarizes device parameters pre-CMR, post-CMR, and at 1-year of follow-up.

**Table 3:** Comparison of device parameters before, after CMR and at 1-year follow-up

	Before CMR	After CMR	At 1-y follow-up	P value
P-wave amplitude (mV)	4.6 ± 4.4	4.6 ± 4.4	3.3 ± 1.0	0.36
R-Wave amplitude (mV)	11.8 ± 4.8	11.8 ± 4.8	11.3 ± 5.1	0.77
Atrial lead impedance (Ohm)	483.3 ± 96.3	453.9 ± 99.05	484.1 ± 108.2	0.78
Ventricular lead impedance (Ohm)	476.2 ± 106.2	497.3 ± 68.8	464 ± 76.2	0.75
Atrial PCT (V@0.4 ms)	0.72 ± 0.29	0.75 ± 0.24	1.08 ± 1.24	0.22
Ventricular PCT (V@0.4 ms)	0.80 ± 0.21	0.82 ± 0.20	0.84 ± 0.30	0.68

### Image quality and diagnostic value of CMR

Among the 68 CMR examinations performed, images were of diagnostic quality in 66 cases (98%).

#### Patients equipped with a MR-conditional PPM

**Fig. 2:**
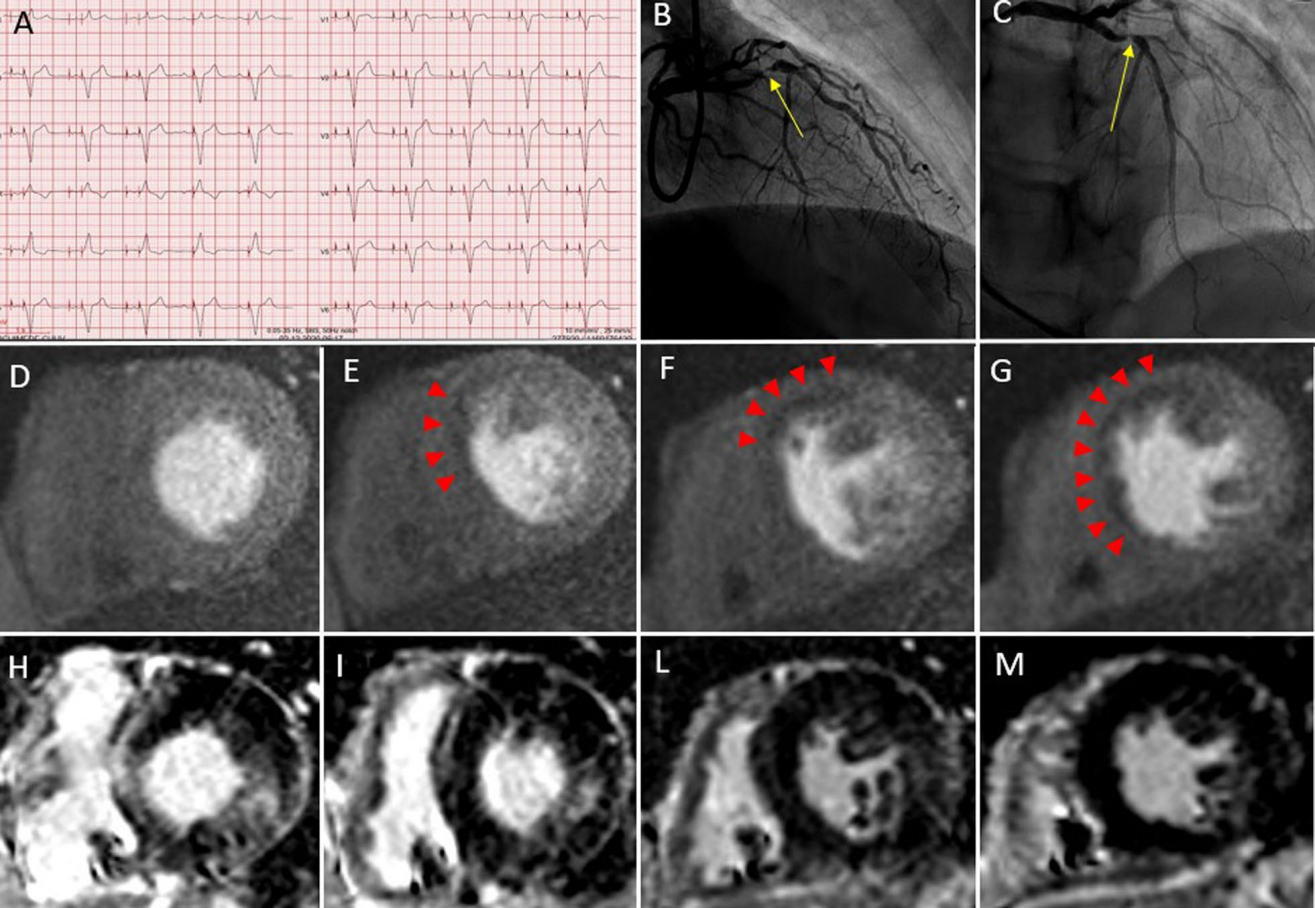
Stress perfusion-CMR in a permanent pacemaker dependent (PPM) patient. A 74-year-old man was referred to stress perfusion CMR due to exertional dyspnea and angor. A double chamber PPM was implanted for a 2-degree atrioventricular block and the patient was PPM-dependent (Panel** A**). X-ray coronary angiography showed a severe stenosis of the proximal portion of the left anterior descending artery (Panel** B**,** C**, yellow arrowed) that was treated by drug-eluting stent implantation. Stress perfusion CMR, performed before x.ray coronary angiography showed an extensive hypoperfusion in the anterior and septal wall (Panel** D**–**G**, red arrowed) without the presence of myocardial scar in late gadolinium (LGE) images (Panel** H**,** I**,** L**,** M**) indicating a positive stress test, consistent with the lesion found in x-ray coronary angiography

In patients with PPMs, 97% of CMR examinations were of good quality leading to a diagnostic examination in 35 cases. In 1 patient stress perfusion was not interpretable due to a Valsalva manoeuvre and a contrast medium injection into a thrombosed vein (this study was repeated successfully 12 days later). Only 2 patients had a “moderate” (grade 2) LGE quality due to respiratory motion (Fig. 2).

#### Patients equipped with a MR-conditional ICD

**Fig. 3:**
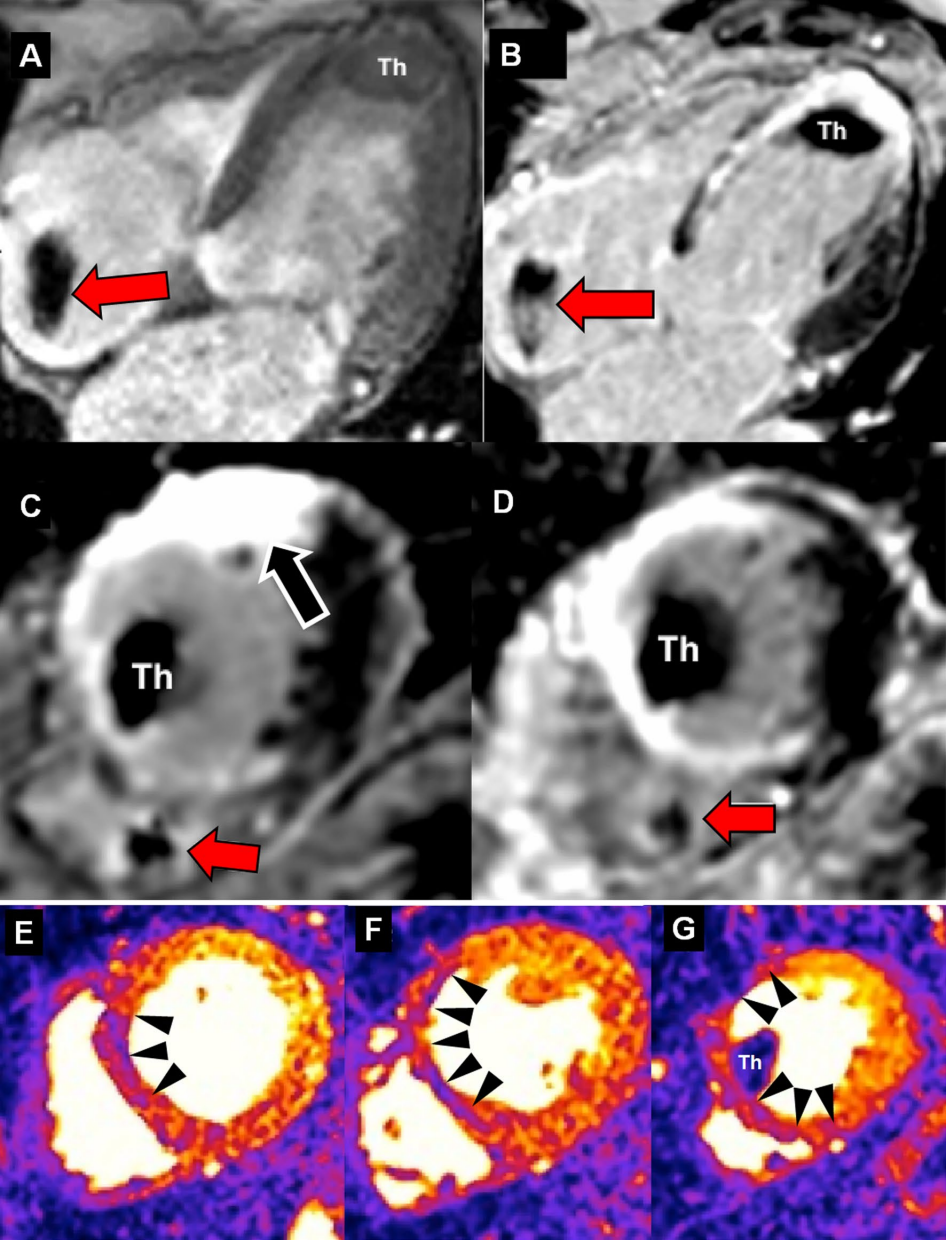
Stress perfusion-CMR in a PPM dependent patient. 67-year-old with known an antero-septal myocardial infarction, was referred to stress perfusion CMR to exclude myocardial ischemia after implantation of a MR-conditional implantable cardiodefibrillator (ICD) for primary prevention. **A** Cine fast-gradient echo acquisition of a 4-chamber view demonstrating a mural thrombus (Th) in the apex and the artifact of the ICD electrode (red arrow). **B** Corresponding standard phase sensitive inversion recovery (PSIR) 4-chamber view with visualization of the post-infarct scar in septal and apical segments as well as the apical thrombus (Th) and the ICD electrode (red arrow). Standard short-axis PSIR in C (with bandwidth of 140 Hz/pixel) and PSIR with increased bandwidth in **D** (300 Hz/pixel) to eliminate the ICD-related artifact on the anterior wall (black arrow in **C**), electrode (red arrow). The mural thrombus (Th) and the antero-lateral scar is visualized. E/F/G: Myocardial perfusion upslope maps of motion-compensated perfusion images in the basal (**E**), mid-ventricular (**F**) and apical (**G**) slices. Visualization of hypoperfused scar (black arrow heads) thereby excluding ischemia in this patient. Hypoperfusion of the mural thrombus in G (Th)

In 2 patients (4%) equipped with a subcutaneous ICD (S-ICD) the cine images were of non-diagnostic quality due to extensive artefacts and consequently, the adenosine stress test was not performed. In the remaining 28 patients (56%) with an MR-conditional transvenous ICD, the perfusion tests were of good quality except for 1 patient who presented with a mild artefact in the anterior LV wall which did not preclude the diagnostic interpretation. LGE was of good quality in 14 patients (50%), of moderate quality in 10 patients (36%) and of poor quality in 4 (14%) patients (Table 4). In all examinations with moderate and poor LGE quality, all artefacts were induced by the devices and projected onto the anterior wall. In only 1 patient with a poor quality CMR examination, artefacts were caused by respiratory motion (Fig. 3).

**Table 4:** Image quality assessment

	Cine								LGE				Perfusion
	SAx		2Ch		3Ch		4Ch		SAx	2Ch	3Ch	4Ch	SAx
	*bSSFP*	*GRE*	*bSSFP*	*GRE*	*bSSFP*	*GRE*	*bSSFP*	*GRE*					
PPM n (%) 36 patients													
Grade 1	36 (100)	–	36 (100)	–	36 (100)	–	36 (100)	–	36 (100)	34 (94)	34 (94)	34 (94)	35 (97)
Grade 2	–	–	–	–	–	–	–	–	–	2 (6)^b^	2 (6)^b^	2 (6)^b^	–
Grade 3	–	–	–	–	–	–	–	–	–	–	–	–	–
Grade 4	–	–	–	–	–	–	–	–	–	–	–	–	1 (3)^a^
Transvenous ICD n (%) 28 patients													
Grade 1	19 (63)	9 (37)	19 (63)	9 (37)	19 (63)	9 (37)	19 (63)	9 (37)	14 (50)	14 (50)	14 (50)	14 (50)	27 (96)
Grade 2	–	–	–	–	–	–	–	–	10 (36)	10 (36)	10 (36)	10 (36)	1 (4)
Grade 3	–	–	–	–	–	–	–	–	4 (14)^c^	4 (14)^c^	4 (14)^c^	4 (14)^c^	–
Grade 4	–	–	–	–	–	–	–	–	–	–	–	–	–
Subcutaneous ICD n (%) 2 patients													
Grade 1	–	–	–	–	–	–	–	–	–	–	–	–	–
Grade 2	–	–	–	–	–	–	–	–	–	–	–	–	–
Grade 3	–	–	–	–	–	–	–	–	–	–	–	–	–
Grade 4	–	2 (100)	–	2 (100)	–	2 (100)	–	2 (100)	–	–	–	–	–

Regarding image quality in patients with Pacemakers: ^&^in 1 patient late gadolinium enhancement (LGE) was graded 2 (“moderate”) due to respiratory motion. ^a^in 1 patient perfusion was not interpretable due to a Valsalva manoeuvre and a contrast media injection into a thrombosed vein. ^b^in 2 exams (6%) the quality was grade 2 (“moderate”) due to respiratory motion

Regarding image quality in patients with ICD: ^c^in 1 exam (3%) quality was grade 3 (“poor”) due to respiratory motion

2Ch, two chamber; 3Ch, three chamber; 4Ch, four chamber; SAx, Short axis

According to the European Society of Cardiology heart failure guidelines [21], CMR revealed preserved LV ejection fraction (LVEF > 49%) in 28 (42%) patients, mid-range impairment (LVEF 40–49%) in 15 (22%) patients, and reduced LV function (LVEF < 40%) in 23 patients (36%).

LGE with a subendocardial or transmural distribution pattern corresponding to post myocardial infarction scarring was present in 23 (44%) patients and was not obscured by device artefacts except in 1 case in which LGE of the anterior LV wall was not interpretable. Post-inflammatory, i.e. sub-epicardial scarring was seen in 2 (4%) patients and nonspecific mediomural fibrosis was present in 4 patients (8%). In those latter cases without myocardial infarction-related scars, no device artefacts were present.

### Adenosine stress perfusion CMR findings

**Fig. 4:**
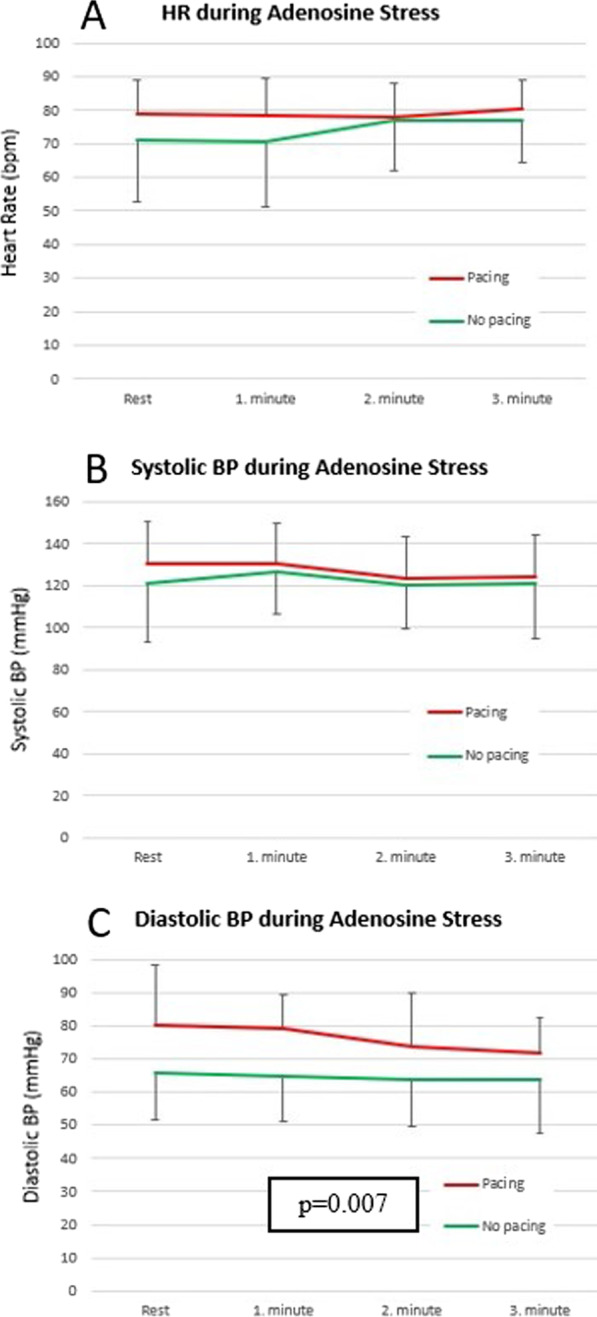
Hemodynamic parameters. Panel **A** heart rate response in patients under pacing (red) or not-paced (green), Panel **B** systolic blood pressure (BP) changes in patients under pacing (red) or not-paced (green), Panel **C** Diastolic BP changes under pacing (red) or not-paced (p = 0.007) (green)

Adenosine stress perfusion CMR was conducted in 68 examinations (all with good cine image quality). During these examinations, no complication during vasodilation occurred and images were of diagnostic quality in 98% of cases. The hemodynamic response during vasodilation is shown in Fig. 4. In non-paced patients, HR increased during vasodilation and thus, stabilized BP, i.e. prevented a BP drop. On the other hand, HR remained unchanged in paced patients, while diastolic BP dropped from 76.0 ± 13.6 mmHg to 68.7 ± 12.5 mmHg (p = 0.007, Fig. 4). During adenosine stress, the splenic switch-off was observed in 78% of the paced patients. Twenty-three patients (36%) reported mild to moderate respiratory symptoms related to adenosine administration.

The stress perfusion CMR test was positive for a perfusion deficit in 20 (31%) patients, and it was located in LGE positive scar tissue in 12 (19%) cases. Significant ischemia, i.e. a perfusion deficit in non-infarcted myocardium > 1.5 segments, was found in 6 cases (9%). Non-significant ischemia (observed in only 1 segment) was found in only 2 patients. These 8 patients were continuously paced in asynchronous mode during the CMR examination.

### Overall clinical outcome

All 6 patients (8%) with a positive stress perfusion CMR test (> 1.5 ischemic segments) underwent invasive coronary angiography that showed critical coronary stenoses. Five of these patients were treated with percutaneous coronary stenting, while 1 patient was conservatively managed with medical treatment due to the small diameter of the stenosed coronary artery. More specifically, in 2 cases of anterior perfusion defects the left anterior descending artery (LAD) was treated, in 1 case of lateral perfusion defect a significant stenosis of the circumflex artery (LCX) was found, and in 3 cases of inferior and infero-lateral perfusion defects a significant stenosis of the right coronary artery was found.

In accordance with the literature [16], both patients with only 1 ischemic segment did not undergo coronary angiography but were treated with medical treatment and remained asymptomatic during follow-up.

In 4 cases (6%) a suspicion of CAD persisted after the negative result of the stress CMR examination and a coronary angiography was performed excluding coronary stenosis in all cases.

A minimum follow-up duration of 6 months was obtained in 57 patients (86%) with a mean follow-up of 3.7 years (range 0.5–7 years). In patient without ischemia, the primary endpoint of MACE occurred in 3 patients (5%): 2 patients experienced a non-ST-elevation myocardial infarction that was treated by percutaneous coronary intervention respectively 2 and 6 years after the negative stress CMR test. One patient with dilated cardiomyopathy and severe LV dysfunction died from decompensated heart failure 5 years after the CMR examination. We also identified 3 non-cardiac deaths (5%). Furthermore, hospitalizations due to decompensated heart failure were recorded in 2 patients (3%) with severely reduced LVEF, respectively 7 months and 2 years after the stress perfusion CMR. In both patients, invasive coronary angiography was repeated and showed no CAD progression. No other cardiac symptoms or cardiac-related hospitalizations were observed during the follow-up period in patients without ischemia.

## Discussion

In the present study we found that adenosine stress perfusion CMR in patients with MR-conditional PPMs and ICDs (1) is safe without competitive cardiac stimulation even when patients were paced during the vasodilator stress test, (2) yields good image quality and hence, provides reliable ischemia detection and (3) does not induce device malfunctioning immediately and at 1 year after the CMR examination.

Recently, the safety of MR-conditional PPM and ICD systems was demonstrated in the setting of CMR examinations aimed at assessing LV and RV morphology and function [9]. However, data on the safety of stress CMR in these patients is scarce, particularly for patients paced during the vasodilator stress test. Klein-Wiele et al. did not report relevant safety issues in a small cohort of 16 patients who were paced during adenosine infusion [13]. Our data on 66 patients, including 30 with implanted ICDs, are confirm this observation, as no pacing-related side effects occurred, i.e. no pacing interference with the intrinsic rhythm, no competitive stimulation, no bradycardia and asystole. In line with the results reported in a testing protocol to identify patients at risk of adenosine-induced deterioration of AV-blocks [11–13], we decided to systematically pace all patients with > 1% of pacing as documented on the pre-CMR device interrogation. With this easily applicable approach, no adverse arrhythmic events occurred during the adenosine stress perfusion CMR examinations.

CMR imaging in patients with implanted devices is notoriously challenging since generator- or lead-related artefacts may affect image quality of the heart and of the large thoracic vessels, hampering interpretation. Quality of stress perfusion CMR images has mainly been reported as good in several series of patients with implanted MR-conditional PPMs [11–13, 22] but none included patients with ICDs. Consequently, our results are in agreement with these reports, and extend the state of knowledge to patients with MR-conditional ICDs. It is noteworthy that a GRE read-out of the perfusion pulse sequence was used, which is known to be less artefact-prone in device patients than a bSSFP read-out [5, 23]. We observed severe artefacts in both patients implanted with subcutaneous ICDs. Interestingly, our observations are in line with the results of the study published by Pezel et al. who reported an image quality graded as good or excellent in 84.1% of segments and diagnostic quality images in 99% of patients with PPMs. ICDs generally have larger generator dimensions, a position closer to the heart and a higher number of implanted leads that may generate larger artefacts, but our experience highlights that good image quality can be achieved in these patients as well.

While the studies mentioned above mainly focused on image quality assessment in patients with PPMs [11–13], the present study also sought to evaluate the diagnostic performance in PPM and ICD patients. A prerequisite for a sensitive detection of ischemia is the induction of maximum myocardial vasodilation in order to optimize the dynamic range of perfusion achieved in highly perfused myocardial territories (subtended by normal coronary arteries) versus hypoperfused territories (subtended by stenosed coronary arteries). In non-paced patients, HR increases during vasodilation to stabilize BP at physiological levels. Consequently, HR increase can be used as an indirect indicator of vasodilation during the stress test. In paced patients, HR is not reliable to assess the vasodilatory effect of adenosine. As observed in the paced patients group with stable HR during vasodilation, the expected BP drop is not compensated. In addition, the presence of a splenic switch-off may also be helpful to assess vasodilation, as it was observed in 78% of paced patients in our study.

In a cohort of 224 patients with PPMs and a 7-year follow-up, Pezel et al. demonstrated that in the absence of inducible ischemia or LGE the cumulative rate of MACE is very low (0.9%) in comparison with patients presenting with either LGE or inducible ischemia, emphasizing on the excellent prognostic value of a negative stress test [13]. Our observations are in line with previous long term results. In our cohort, myocardial ischemia was detected in 8 patients, among whom 6 had significant ischemia > 1.5 ischemic segments [16], prompting coronary angiography which showed significant coronary stenosis in all cases. In the other 2 patients the burden of ischemia was limited (< 1.5 ischemic segments). They were treated with medication and no MACE occurred during follow-up. Notably, inducible ischemia detected in stress perfusion CMR correlated in all cases with the results of the coronary angiography. Conversely, in 4 ischemia-negative patients on stress perfusion CMR, clinical suspicion persisted, and coronary angiography was subsequently performed, confirming the absence of coronary stenosis in all cases. In the absence of ischemia on the perfusion CMR scan, coronary angiography was not performed, and patients were followed up over a mean period of 3.7 years. No MACE occurred in the 57 ischemia negative patients during the first 2 years following the CMR examination. A non-ST elevation myocardial infarction (NSTEMI) was recorded in 2 ischemia-negative patients later during follow-up (after 48 and 84 months), which was treated with percutaneous coronary intervention. A third ischemia-negative patient with known dilated cardiomyopathy and CAD died of heart failure. Moreover, 2 patients were hospitalised due to heart failure and a coronary angiography ruled out CAD progression in both patients. These results suggest a high specificity for ischemia-positive patients (6 of 6 patients correctly diagnosed) combined with a good sensitivity over 2 years (no MACE occurred in ischemia-negative patients during this period) and the MACE rate was 1.6% per year over the entire follow-up of 3.7 years.

Finally, the present study showed no clinically significant alterations of device functioning immediately after CMR and at 1 year of follow-up further encouraging the use of adenosine stress CMR perfusion imaging in patients with MR-conditional PPMs and ICDs [5, 23].

## Limitations

The main limitations of this study is the limited sample size and the retrospective nature of the analysis. Moreover, rest perfusion was not acquired, thus the analysis were made only for stress perfusion images. Future prospective studies with larger sample sizes are warranted to confirm our findings. Some device models such as subcutaneous ICDs (n = 2) or leadless PPMs (n = 1) were under-represented in the study and conclusions should be derived with caution for these devices. Our limited data (n = 2) suggest image quality is adversely impacted in patients with a subcutaneous ICDs.  Leadless ICDs are also being investigated and remain unexplored with regards to vasodilator CMR.

## Conclusions

In this series of patients with MR-conditional transvenous PPMs and ICDs, adenosine stress-CMR was safe in both, paced and non-paced patients. It provided good image quality with reliable ischemia detection. No device malfunctions were observed immediately and at 1 year after the CMR examination.

## Supplementary Information

**Additional file 1: Table S1. **Detailed sequences characteristics.

## Data Availability

The datasets used and analysed during the current study are available from the corresponding author on reasonable request.

## Abbreviations

AV: Atrioventricular
BP: Blood pressure
bSSFP: Balanced steady state free precession
CAD: Coronary artery disease
CMR: Cardiovascular magnetic resonance
cTn: Cardiac troponin
ECG: Electrocardiogram
GBCA: Gadolinium based contrast agent
GRE: Gradient echo
HR: Heart rate
ICD: Implantable cardioverter defibrillator
LAD: Left anterior descending coronary artery
LCX: Left circumflex coronary artery
LGE: Late gadolinium enhancement
LV: Left ventricle/left ventricular
LVEF: Left ventricular ejection fraction
MACE: Major adverse cardiovascular events
MR: Magnetic resonance
NSTEMI: Non-ST elevation myocardial infarction
PPM: Permanent pacemaker
PSIR: Phase sensitive inversion recovery
RA: Right atrium/right atrial
RV: Right ventricle/right ventricular
S-ICD: Subcutaneous implantable cardioverter defibrillator
