# Supplementary material for: Feasibility of adenosine stress cardiovascular magnetic resonance perfusion imaging in patients with MR-conditional transvenous permanent pacemakers and defibrillators
Source: J Cardiovasc Magn Reson. 2022 Jan 13;24:9. doi: 10.1186/s12968-021-00842-0 (PMC8756706; doi:10.1186/s12968-021-00842-0)
Supplement: Supplementary file 1 — Additional file 1: Table S1. Detailed sequences characteristics. [file 12968_2021_842_MOESM1_ESM.docx]

| **CMR Protocol** | **Characteristics** |
| --- | --- |
| **Scanners** | 1.5T Aera; Siemens-Healthineers, Erlangen, Germany or  1.5 T Sola; Siemens-Healthineers |
| **Cine Imaging**  **(bSSFP)** | Balanced steady state free precession  Slices :12  Number of frame:25(phase)  FOV: 300x244 mm  Matrix:256x146  Slice thickness: 8 mm  Slice gap: 2 mm  TR /TE: 49/^1.28 ms  Flip-angle: 80°  Phase oversampling: 50%  Slice oversampling:0  Acceleration:Ipat, Grappa2 |
| **Cine Imaging**  **(Fast Gradient Echo)** | Fast gradient echo  Slices : 12  Number of frame:25(phase)  FOV: 300x244mm  Matrix:256x146  Slice thickness: 8mm  Slice gap: 2mm  TR /TE: 50/3.45 ms  Flip-angle: 15  Phase oversampling:50%  Slice oversampling:0  Acceleration: Ipat Grappa2 |
| **Contrast Agent / Dosage** | Gadobutrol (Gadovist®, Bayer Healthcare, Berlin, Germany) of 0.2 mmol/kg IV |
| **Stress Perfusion Test** | Fast Gradient Echo  4 slices  Number of frame: (83segements)  FOV: 260x295mm  Matrix:208x139  Slice thickness: 8mm  Slice gap: 1.6-8mm  TR /TE: 250/1.2ms  Flip-angle:12  Phase oversampling: 19%  Slice oversampling:0  Acceleration: Ipat Grappa2 |
| **Late Gadolinium Enhancement** | 2D segmented Phase-sensitive inversion recovery Gradient Echo  FOV: 340x276 mm  Matrix:256x156  Slice thickness: 8 mm  Slice gap: 2 mm  TR /TE: 414/3.2 ms  Flip-angle: 25°  Phase oversampling:0  Slice oversampling:0  Acceleration:0 |

Table S1: detailed sequences characteristics.
